# Comparative analysis of the quality characteristics and flavor volatiles of Lueyang black-bone chicken meatballs cooked via different methods

**DOI:** 10.3389/fnut.2025.1629738

**Published:** 2025-07-23

**Authors:** Wen Su, Shibo Zhao, Jinfeng Zhou, Linjie Xi, Wengang Jin, A. M. Abd El-Aty

**Affiliations:** ^1^Qinba State Key Laboratory of Biological Resources and Ecological Environment (Incubation), Shaanxi University of Technology, Hanzhong, China; ^2^School of Bioscience and Engineering, Shaanxi University of Technology, Hanzhong, China; ^3^Department of Pharmacology, Faculty of Veterinary Medicine, Cairo University, Giza, Egypt; ^4^Department of Medical Pharmacology, Faculty of Medicine, Atatürk University, Erzurum, Türkiye

**Keywords:** black-boned chicken, steaming, boiling, air-frying, GC–IMS, volatile compounds

## Abstract

Chicken meatballs are well received by consumers. This study evaluated the physicochemical properties and sensory attributes of Lueyang black-bone chicken meatballs prepared via three different cooking methods—steaming, boiling, and frying—and further analyzed their volatile compound profiles via gas chromatography–ion mobility spectrometry (GC–IMS). Compared with the steamed and boiled samples, the fried meatballs presented the highest sensory scores and greater hardness, adhesiveness, and chewiness (*p* < 0.05). Moreover, the color parameters [L*(34.67 ± 1.63), a*(6.89 ± 0.07), b*(15.12 ± 0.79)] of the fried samples differed notably from those of the other samples (*p* < 0.05), indicating the effect of thermal processing on their appearance. A total of 42 volatile organic compounds (VOCs), primarily ketones (31.92–47.55%), acids (17.57–24.33%), and esters (8.53–16.67%), were identified. OPLS-DA with VIP analysis (VIP > 1.0, *p* < 0.05) revealed 17 differential VOCs, with (E)-3-hexen-1-ol, hexanal, and ethyl 2-methylpropanoate significantly upregulated in fried samples (log2FC > 1). Overall, fried Lueyang black-bone chicken meatballs presented more favorable quality attributes. These results may offer valuable insights for the value-added development of Lueyang black-bone chicken meatball products.

## 1 Introduction

Lueyang black-bone chickens constitute a distinctive poultry breed native to Lueyang County, Shaanxi Province, China. Black-bone chickens exhibit distinct physical characteristics: their external body parts and some organs tend to be gray or black, which is caused by the presence of melanin (1, 2). It is known for its delicious meat and rich nutritional content, and it also exhibits robust free-range rearing capabilities (3). The breed is large and nutritionally rich, with firm meat that contains a high concentration of trace elements necessary for the human body and has significant medicinal value (4). Compared with regular chicken meat, black-bone chicken meat is distinguished for having less fat and a higher concentration of essential amino acids and unsaturated fatty acids (5), making it an ideal ingredient for healthier culinary options. As consumers increasingly seek out foods with health benefits, products such as low-fat, healthy meatballs, which can incorporate such nutritious ingredients, have become a popular trend (6, 7). Researchers have conducted studies on this topic in the fields of molecular biology and genetics (8, 9), in addition to meat quality, amino acids, and transcriptomics under different rearing systems (4), as well as on the metabolomics of the meat (7) along with volatile compounds (VOCs) in distinct chicken tissues (5). Consequently, there is a strong demand for the processing and development of Lueyang black-bone chicken products.

Currently, most commercially available meatballs are made from pork, beef, or mutton and typically contain high fat content and starch, which do not align with modern consumer preferences for healthier options (10). Chickens, which are high in protein, low in fat, easily absorbed by the body, and able to enhance immunity, are suitable for developing meatballs designed for fat reduction and muscle gain (11). With the growing production and rapid expansion of Lueyang black-bone chicken farming, developing meatball products can enhance product diversity and broaden consumer options in the market. Cooking is an essential method for producing meat products, developing flavors, and ensuring food safety through heating (12). Common cooking methods include steaming, boiling, frying, and smoking, which impact basic properties, such as flavor and taste, of meat products, thereby affecting consumer preferences (13, 14). Different cooking methods and degrees of doneness can significantly alter the internal structure and physicochemical properties of meat products, ultimately influencing their digestibility (15, 16). To evaluate these changes, researchers commonly assess parameters such as hardness, texture, color variation, cooking yield, and sensory attributes to objectively determine product quality. Owing to differences in heat transfer media, as well as variations in cooking time and temperature, the extent of thermal reactions during processing also differs (17, 18), thereby impacting the generation of volatile organic compounds (VOCs).

Recently, GC–IMS has emerged as an approach for detecting trace VOCs. This authenticity identification technology has several advantages: rapid analysis, high sensitivity, and good selectivity; no need for pretreatment and enrichment, simplifying sampling methods and accurately reflecting food components; operation at atmospheric pressure without the need for a vacuum, reducing operational costs; and the detection process does not rely on organic solvents, making it environmentally friendly and pollution free (19, 20). The flavor fingerprint graphs produced allow for the visual analysis of trace component differences between samples and have been utilized in the detection of various meat-based products, such as *Crassostrea gigas* (21), lamb (22), and meatballs (23).

Despite the growth of Lueyang black-bone chicken farming, deep processing and value-added product development remain limited. Our earlier research explored the steaming process of black-bone chicken meat (19) and analyzed volatile flavor compounds across different anatomical cuts (5). However, the quality characteristics and flavor profiles of Lueyang black-bone chicken meatballs cooked via different methods are still unclear. Therefore, we assessed and compared the sensory characteristics, texture, color, quality change rates, and volatile organic compounds (VOCs) of black-bone chicken meatballs cooked by boiling, steaming, and frying, which could provide guidance for the development and quality control of processed meatball products.

## 2 Materials and methods

### 2.1 Materials and reagents

Freshly prepared Lueyang black-bone chicken meatballs were purchased from Shaanxi Longjia Agricultural Technology Co., Ltd. (Hanzhong, China). To maintain sample freshness and uniformity, all the meatballs were analyzed on the day of purchase without undergoing freezing. Before being subjected to sensory evaluation and GC–IMS analysis, the samples were stored at 4 ± 1°C for a maximum of 6 h. The ingredients were sourced from a local supermarket and included black-bone chicken meat, spices, food additives (monosodium glutamate, sodium tripolyphosphate, sodium hexametaphosphate, sodium hexametaphosphate, carrageenan), and chicken powder seasoning, along with starch, soybean protein, edible salt, and white sugar. Standard n-ketones were purchased from Guoyao Chemical Co., Ltd. (Shanghai, China).

### 2.2 Preparation of Lueyang black-bone chicken meatballs by different cooking methods

Chicken meatballs were prepared according to previous method Aydemir et al. (24, 25), with slight modifications. Uniform black-bone chicken meatball samples were selected for preliminary testing. On the basis of the preliminary experiment and sensory evaluation, the optimal texture of the meatballs was achieved after 6 min of cooking. Prolonged cooking beyond this duration adversely affects the color and texture of meatballs while also causing a loss or destruction of nutritional components. Under these conditions, the meatballs demonstrated optimal overall quality, reflected by the highest sensory evaluation scores and favorable instrumentally measured color and texture parameters. Therefore, the single-factor experimental time was set to 6 min. After thawing and weighing, black-bone chicken meatballs were prepared via three different cooking techniques. For the boiling method, the meatballs were subjected to boiling water at 2100 W for 6 min, cooled, air-dried until moisture-free, and reweighed. For steaming, the samples were steamed at the same power for 6 min (C22-RT22E01, Midea Group Co. Ltd., China), followed by cooling and air drying before being weighed again. For frying, the meatballs were coated with rapeseed oil, air dried at 180°C for 6 min, and then reweighed. For each cooking method, three meatballs were prepared and analyzed, each with an approximate radius of 1.5 ± 0.2 cm. During frying, the meatballs were cooked over medium heat for 6 min in total—approximately 3 min per side—while being turned regularly to ensure uniform cooking.

### 2.3 Sensory evaluation

Sensory evaluation of meatballs prepared through different cooking methods followed the standards outlined in SB/T 10610–2011. The sensory evaluation criteria for black-bone chicken meatballs (Supplementary Table S1) included assessments of color, taste, texture, and juiciness. Twenty trained sensory panelists (10 males and 10 females, aged 20–22 years), all undergraduate students, were recruited to evaluate the meatballs on the basis of the specified quality attributes.

### 2.4 Color difference analysis

An SR-62 colorimeter (3nh Technology Co., Shenzhen, China) was used according to the method of Gui et al. (26). Three parallel groups were measured for each sample, and six points per group were analyzed. The results were averaged. For the meatballs prepared via different cooking methods, the surface water and oil of the black-bone chicken meatballs were absorbed via absorbent paper. A portable colorimeter was used to quantify the L* (lightness), a* (redness), and b* (yellowness) values.

### 2.5 Texture analysis

In accordance with the methods of Li et al. (27), texture was determined via a Brookfield CT3 texture analyzer (Brookfield, United States). The parameters used were as follows: TA39 probe, with a trigger point set to 5.0 g, a testing speed of 1.0 mm/s, and 2 testing cycles per sample, with each sample repeated four times to obtain the average value. A force–time curve was generated on the basis of these measurements. From this curve, hardness, elasticity, cohesiveness, and chewiness were analyzed. Six measurements were performed for each sample group.

### 2.6 Measurement of quality change rates during steaming, boiling, and frying

Steaming and boiling: Chicken meatballs were weighed before (W_1_) and after steaming or boiling (W_2_) and cooled, and the gain–loss percentage was calculated as (_W2–W1_)/W_1_ × 100%.

Frying: The meatballs were weighed prefrying (G_1_) and postfrying (G_2_) after draining excess oil and cooling. The frying gain–loss percentage was determined via the formula (G_2_ – G_1_)/G_1_ × 100%.

### 2.7 GC–IMS detection of volatile organic compounds (VOCs)

Two grams of black-bone chicken meatball samples were accurately weighed and placed into a 20 mL headspace vial. The incubation temperature was 60°C; the incubation time was 20 min; the sample volume was 500 μL; the splitless injection rate was 500 r/min; and the injection needle temperature was 85°C. Each sample group was measured in triplicate. The specific operation and parameters of the GC–IMS analysis were consistent with those of our previous paper (5). GC separation was carried out using an MXT-5 column at 60°C with high-purity nitrogen (≥99.99%) as the carrier gas over a 20-min run. The initial flow rate of 2.0 mL/min was maintained for 2 min, then increased linearly to 10 mL/min over the next 10 min, and further increased to 100 mL/min by the end of 20 min. Ion mobility spectrometry (IMS) was performed using a detector maintained at 45°C with a nitrogen flow rate of 150 mL/min, and analysis continued for 30 min. Prior to sample testing, calibration standards comprising six C4–C9 n-ketones were analyzed under identical chromatographic conditions to establish retention index (RI) versus retention time (RT) calibration curves. These ketones, with RIs set at 100 times their respective carbon numbers, were used as reference markers. VOCs in different meat cuts were identified and relatively quantified on the basis of drift time (DT) and RI values via the IMS drift time library and NIST 2010 database. The peak volume of each identified compound was also calculated to estimate its relative abundance.

### 2.8 Statistical analysis

All the data are presented as the means ± standard deviations (*n* = 3). Volatile organic compounds (VOCs) were identified via the NIST 2020 database in combination with a custom-built IMS library. Spectral differences were visually compared via the Reporter plugin, whereas quantitative differences in VOCs among samples were assessed via fingerprint analysis via the Gallery Plot plugin. Bar charts representing the relative abundances of individual components were generated via Excel 2020. Multivariate analyses, including principal component analysis (PCA) and orthogonal partial least squares-discriminant analysis (OPLS-DA), were performed via the BioDeep Tool Assistant^1^, which also facilitated the creation of variable importance in projection (VIP) plots and clustering heatmaps. One-way analysis of variance (ANOVA) was conducted to assess group differences, with Duncan’s multiple range test applied for *post hoc* comparisons. The statistical significance was set at *p* < 0.05.

## 3 Results and discussion

Different heating approaches influence the sensory evaluation, color, and texture of meat products. Moreover, heat treatment promotes the generation of a wide range of volatile compounds through Maillard reactions and lipid oxidation, thereby significantly enhancing the flavor complexity of black-bone chicken meatballs and imparting a more appealing aroma and taste to the product (28, 29). However, inappropriate cooking parameters may lead to the formation of heterocyclic amines and volatile aldehydes, resulting in off-flavors and potential health risks (30). In addition, high-temperature treatment can cause nutrient degradation in meatballs; for example, studies have shown that frying decreases the levels of EPA and DHA (31). Therefore, determining a suitable heating method is crucial for both the retention of nutrients and the development of desirable flavor compounds. The following sections provide a systematic analysis of the comprehensive effects of steaming, boiling, and frying on the quality attributes of Lueyang black-bone chicken meatballs.

### 3.1 Sensory evaluation of Lueyang black-bone chicken meatballs prepared via different cooking methods

**Figure 1:**
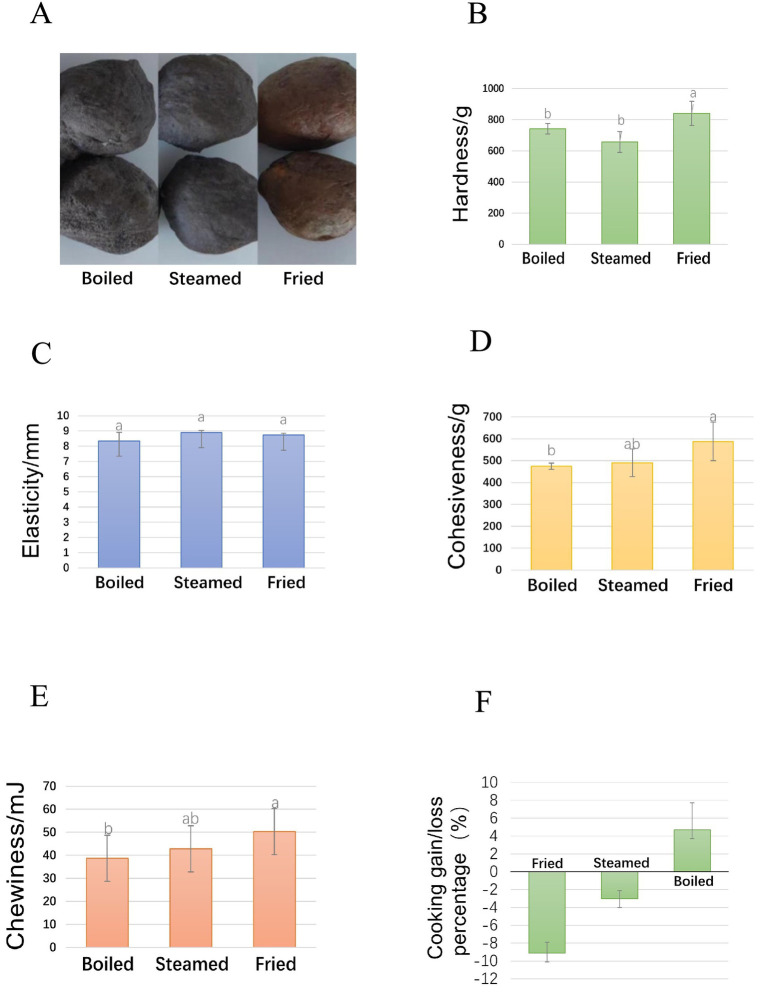
Lueyang black-boned chicken meatballs cooked by boiling, steaming, and frying methods: **(A)** appearance, **(B)** hardness, **(C)** elasticity, **(D)** cohesiveness, **(E)** chewiness, and quality change rate **(F)**.

Sensory evaluation is the process in which evaluators use their normal visual, olfactory, and taste perception abilities to comprehensively analyze and assess the quality factors of samples, including appearance, color, and flavor (32). Compared with boiled and steamed chicken meatballs, fried black-bone chicken meatballs presented a superior color, appearance, and taste (Figure 1A). This is attributed to the Maillard reaction that occurs during air-frying, which enhances the color, flavor, and sensory characteristics of the meatballs. Although traditional deep-fat frying is considered less healthy, this study revealed that air-fried black-bone chicken meatballs presented superior sensory qualities due to reduced oil absorption. In contrast, simpler methods, such as boiling and steaming, resulted in minimal changes in texture and flavor. Given the same cooking duration, frying notably enhanced both color and flavor, which was reflected in higher sensory evaluation scores (Supplementary Table S2).

### 3.2 Color difference analysis of Lueyang black-bone chicken meatballs prepared via different cooking methods

Color plays a significant role in evaluating the quality of meat and meat products and is typically evaluated through L*, a*, and b* values (10). Compared with boiled and steamed samples, the fried black-bone chicken samples presented significantly greater L*, a*, and b* values (*p* < 0.05) (Table 1), most likely because the Maillard reaction occurs at high temperatures during frying, and the L*a*b* values are associated primarily with the formation of brown pigments called melanoidins (29). The L* value of the boiled samples was marginally greater than that of the steamed samples, possibly because the boiling process, where the meatballs are fully submerged in water, absorbs a considerable amount of moisture, leading to a higher surface moisture content and better gloss. In contrast, steaming uses water vapor to cook meatballs, which does not impart the same level of surface gloss. The L*, a*, and b* values of the boiled and steamed samples were not different (*p* > 0.05), but the fried samples presented relatively high a* and b* values, indicating that frying increased the redness and yellowness of the meatball color (*p* < 0.05) (Figure 1A). The reasons for different colors include various external and internal factors, such as additives, proteins, myoglobin, moisture, and cooking methods, among which myoglobin primarily controls the color of raw and cooked meat (33, 34). The present air-fried meatballs might undergo the Maillard reaction at high temperatures, which significantly alters the color and aroma of the meatballs.

**Table 1:** Color difference values of different cooking methods of Lueyang black-boned chicken meatballs.

Cooking method	Lightness (L* value)	Redness (a* value)	Yellowness (b* value)
Boiled	32.35 ± 1.29^b^	2.37 ± 0.23^b^	5.54 ± 0.47^b^
Steamed	31.22 ± 0.16^b^	2.23 ± 0.16^b^	5.09 ± 0.06^b^
Fried	34.67 ± 1.63^a^	6.89 ± 0.07^a^	15.12 ± 0.79^a^

Different lowercase letters with superscripts in the same row denote significant differences between samples (*p* < 0.05).

### 3.3 Texture analysis of Lueyang black-bone chicken meatballs cooked via different methods

Texture is pivotal in evaluating food quality and consumer acceptance, providing an objective measure of meatball quality (35). The hardness, elasticity, adhesiveness, and chewiness results are shown in Figure 1. As shown in Figure 1B, the fried meatballs were significantly harder than the other groups were (*p* < 0.05). The hardness of the fried meatballs reached a maximum value of 840.2 g. Elasticity, which refers to the height at which the food can recover between the first bite and second bite, did not significantly differ among the groups (Figure 1C), with all values being approximately 8.5 mm. Adhesiveness quantifies the energy needed to separate semisolid foods into a swallowable form. Compared with steamed and boiled meatballs, frying increased the adhesiveness (Figure 1D) and chewiness (Figure 1E) of the black-bone chicken meatballs, reaching maximum values of 587.9 g for adhesiveness and 50.39 mJ for chewiness. These results indicate that frying can significantly improve the hardness, adhesiveness, and chewiness of black-bone chicken meatballs. Moreover, frying promotes the formation of a crispy exterior, a crucial factor in enhancing textural perception. As moisture evaporates during frying, a porous surface structure develops, while interactions between proteins and starches result in a cohesive biopolymer network. This network reinforces the crust, which fractures under chewing pressure, creating a satisfying crisp sensation. These structural modifications not only improve the mechanical attributes of the product but also increase consumer acceptance by delivering a firmer and more appealing mouthfeel (36, 37).

### 3.4 Quality change rates of Lueyang black-bone chicken meatballs cooked via different methods

As shown in Figure 1F, the rate of change in the quality of black-bone chicken meatballs cooked via different methods (boiled, steamed, and fried) tended toward both loss and gain rates. The loss rate is indicated on the lower part of the horizontal axis, whereas the gain rate is represented on the upper part. For boiling, the weight of the meatballs increased. This is because the meatballs were fully immersed in water during boiling, absorbing a large amount of moisture, and when the meatballs were cooled to room temperature, the absorbed moisture had not fully evaporated, leaving some moisture inside the meatballs. During steaming, the meatballs were cooked with steam vapor. During the steaming process, some moisture entered the meatballs, but the moisture content was lower than that during boiling. The weight loss in steamed meatballs was approximately 3%, which was negligible. During frying, high temperatures cause the moisture in the meatballs to evaporate. The moisture content was significantly reduced, resulting in the greatest weight loss, approximately 9%. However, weight loss tended to be lower in fried meatballs than in boiled meatballs. This occurred because, during frying, moisture evaporated at a relatively high rate, but a crust formed on the surface of the meatballs, which effectively reduced moisture loss and enhanced the water retention ability of the meatballs. The cooking process often affects the juiciness of meat products, which in turn impacts their water-holding capacity (38). Studies on beef have demonstrated a negative correlation between juiciness and cooking loss, indicating that greater cooking losses lead to a decrease in juiciness (39).

### 3.5 GC–IMS analysis results

#### 3.5.1 Comparison of VOC differences in Lueyang black-bone chicken meatballs cooked via different methods

**Figure 2:**
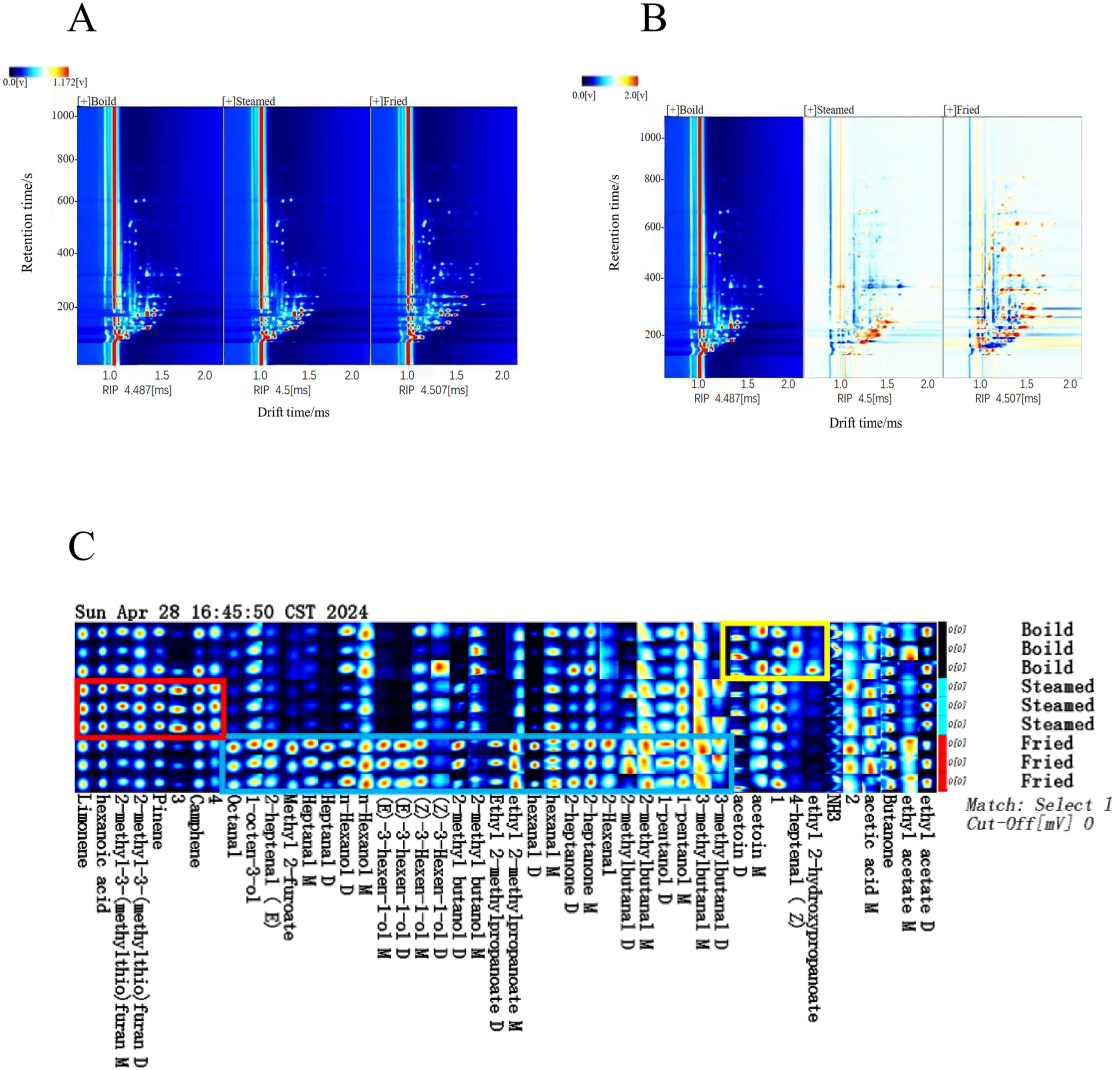
VOCs of black-bone chicken meatballs: **(A,B)** GC–IMS 2D overview spectra and difference spectra; **(C)** fingerprint.

Figure 2 shows the GC–IMS 2D overview spectra and difference spectra for the VOCs of black-bone chicken meatballs prepared by boiling, steaming, and frying. Variations in VOC composition and concentration can be visualized through analysis of peak occurrence and color intensity. The *y*-axis denotes the retention time from gas chromatography, whereas the *x*-axis represents the relative ion mobility time (40). Compared with the boiled and steamed samples, the fried samples contained higher levels of volatile compounds (Figure 2A). The differences among the samples are compared in Figure 2B. This comparison highlights the distinct volatile profiles, emphasizing the higher concentration and variety of VOCs in the fried meatballs than in the other cooking methods.

#### 3.5.2 Fingerprint spectra of Lueyang black-bone chicken meatballs cooked via different methods

To directly determine the differences in the contents of volatile organic compounds (VOCs) in Maillard reaction products at different temperatures, volatile fingerprint spectra were constructed and analyzed. All the peaks were selected, and GalleryPlot was used to plot the volatile compound fingerprint spectrum. Parallel experiments were conducted on black-bone chicken meatballs cooked via different methods, and each parallel group was measured three times, yielding the GC–IMS spectra of VOCs in the meatballs cooked via different methods (20). The horizontal axis indicates the three parallel black-bone chicken meatball samples from different cooking methods, whereas the vertical axis shows the presence of the same VOCs in each cooking method (Figure 2C). The VOCs in meatballs are relatively complex and include aldehydes, esters, ketones, alcohols, alkenes, acids, and furan organic compounds. Significant differences in VOCs are observed across the different cooking methods (as indicated by the blue, red, and yellow boxes). The fried samples had the most flavor compounds, likely due to their high temperatures. Among these methods, the frying method resulted in the highest levels of compounds such as hexanal, heptanal, 2-hexenal, 2-methylbutanal, (E)-3-hexen-1-ol, and (Z)-3-hexen-1-ol. However, a few compounds, such as acetoin, 4-heptenal-(Z), and ethyl 2-hydroxypropanoate, were more abundant in the boiled samples (Figure 2C). Compared with high-temperature methods, this cooking method yields meat with improved visual appeal and tenderness while preserving desirable texture, juiciness, and succulence (41, 42).

Previous studies have reported that different cooking methods influence not only food quality but also food safety, particularly through the formation of Nε-(carboxymethyl) lysine (CML), a marker of advanced glycation end products. Assar et al. (43) reported that CML levels were significantly higher in fried ground beef (11.2 mg/kg) than in boiled beef (5.02 mg/kg), with CML accumulation closely associated with the extent of lipid oxidation. This is largely due to aldehydes generated during lipid oxidation facilitating reactions between lysine residues and reactive compounds such as glyoxal (GO), resulting in structural alterations to proteins and the formation of harmful substances. Additionally, dicarbonyl compounds formed during oxidation can further react with lysine, accelerating the production of GO—the primary precursor of CML—and thereby increasing CML levels (24, 25). While this study focused on the quality attributes and volatile profiles of black-bone chicken meatballs prepared via three cooking methods, future research should investigate the formation of such harmful compounds and explore potential correlations with identified volatiles.

#### 3.5.3 Qualitative analysis of VOCs in Lueyang black-bone chicken meatballs

Through analysis of the retention and migration times of major flavor compounds, VOCs from different cooking methods were qualitatively analyzed via identification via the GC–IMS database. To further examine the changes in VOCs in boiled, steamed, and fried samples, the flavor analyzer NIST gas–phase retention index and IMS migration time database were utilized (5). A total of 42 VOCs, including 12 aldehydes, 11 alcohols, 6 esters, 6 ketones, 3 alkenes, 2 acids, and 2 furans, were identified across the three cooking methods (Table 2).

**Table 2:** Volatile organic compounds identified across the three cooking methods in Lueyang black-bone chicken meatballs.

	Compound	Boiled	Steamed	Fried
Acids	Hexanoic acid	787.84 ± 98.53b	957.79 ± 24.91a	805.11 ± 95.84ab
	Acetic acid	3150.8 ± 196.81a	2742.71 ± 149.69a	3011.44 ± 719.57a
Alcohols	1-octen-3-ol	1088.57 ± 22.06ab	981.78 ± 35.06b	1289.75 ± 185.98a
	n-hexanol-D	687.21 ± 188.29a	282.78 ± 132.01b	783.47 ± 177.77a
	n-hexanol-M	1375.95 ± 105.62a	1031.35 ± 188.69b	1436.66 ± 162.57a
	(E)-3-hexen-1-ol-M	84.02 ± 6.17b	68.18 ± 1.11b	617.13 ± 21.85a
	(E)-3-hexen-1-ol-D	15.95 ± 8.16b	9.79 ± 0.61b	189.83 ± 23.51a
	(Z)-3-hexen-1-ol-M	287.67 ± 127.98b	347.02 ± 53.15b	553.96 ± 17.46a
	(Z)-3-hexen-1-ol-D	113.51 ± 73.52a	97.01 ± 29.52a	87.39 ± 15.44a
	1-pentanol-M	505.78 ± 84.04a	598.32 ± 47.56a	541.55 ± 18.58a
	1-pentanol-D	172.16 ± 59.34b	257.52 ± 28.14a	272.45 ± 22.16a
	2-methyl-1-butanol-M	3197.79 ± 672.86a	1821.43 ± 390.87b	1486.76 ± 707.24b
	2-methyl-1-butanol-D	484.1 ± 348.77b	1452.94 ± 487.64b	2794.22 ± 961.29a
Aldehydes	Octanal	174.02 ± 39.87b	144.9 ± 3.99b	484.93 ± 82.24a
	2-heptenal-(E)	109.31 ± 20.05b	119.88 ± 6.9b	314.34 ± 71.79a
	Heptanal-D	38.42 ± 2.9b	36.7 ± 2.48b	201.4 ± 84.4a
	Heptanal-M	232.04 ± 12.91b	195.73 ± 9.1b	583.98 ± 115.41a
	Hexanal-M	705.91 ± 230.77b	855.16 ± 86.14ab	1046.54 ± 11.19a
	Hexanal-D	379.53 ± 181.31b	494.65 ± 91.17b	2918.14 ± 1051.18a
	4-heptenal-(Z)	206.09 ± 115.86a	118.39 ± 17.94a	95.87 ± 12.7a
	2-methylbutanal-M	454.82 ± 40.32b	479.46 ± 24.53ab	525.18 ± 20.25a
	2-methylbutanal-D	228.7 ± 84.87b	634.75 ± 258.49ab	993.48 ± 491.44a
	3-methylbutanal-M	476.8 ± 70.83a	522.67 ± 67.58a	543.62 ± 47.14a
	3-methylbutanal-D	307.94 ± 93.64a	957.06 ± 444.38a	1003.04 ± 435.75a
	2-Hexenal	42.1 ± 3.46b	36.5 ± 1.22b	90.59 ± 24.07a
Esters	Methyl 2-furoate	110.51 ± 27b	82.8 ± 6.64b	337.82 ± 105.44a
	Ethyl 2-hydroxypropanoate	499.94 ± 321.63a	210.49 ± 57.62a	161.25 ± 22.76a
	ethyl 2-methylpropanoate-D	118.08 ± 61.15b	68.53 ± 17.29b	650.31 ± 101.67a
	ethyl 2-methylpropanoate-M	328.35 ± 16.98b	331.27 ± 57.84b	655.85 ± 26.84a
	Ethyl acetate-D	5853.68 ± 2770.13a	5864.3 ± 616.97a	5267.79 ± 1043.85a
	Ethyl acetate-M	305.51 ± 192.41a	240.93 ± 40.35a	408.82 ± 110.02a
Furans	2-methyl-3-(methylthio)furan-M	359.07 ± 69.89ab	443.98 ± 32.79a	290.01 ± 29.79b
	2-methyl-3-(methylthio)furan-D	402.49 ± 47.6b	500.63 ± 24.79a	354.87 ± 24.93b
Ketones	1-octen-3-one	297.88 ± 285.67a	151.25 ± 10.8a	181.22 ± 84.92a
	2-heptanone-D	204.3 ± 111.39ab	133.27 ± 8.37b	272.46 ± 26.01a
	2-heptanone-M	488.55 ± 91.04ab	464.99 ± 41.56b	610.35 ± 43.12a
	Butanone	8926.53 ± 288.04c	9481.81 ± 79.95b	10047.41 ± 270.07a
	Acetoin-D	11174.6 ± 1990.04a	7718.28 ± 1643.54ab	6382.49 ± 1920.61b
	Acetoin-M	716.3 ± 310.56a	765.05 ± 68.93a	691.27 ± 97.58a
Terpenes	Pinene	520.16 ± 202.72a	683.09 ± 37.07a	470.42 ± 26.81a
	Camphene	696.85 ± 352.57a	946.85 ± 50.12a	670.43 ± 30.25a
	Limonene	685.21 ± 208.95a	882.08 ± 21.91a	703.88 ± 50.97a

The suffix D in the table represents dimers of volatile organic compounds, whereas M represents monomers. Different lowercase letters with superscripts in the same row denote significant differences between samples based on ANOVA (*p* < 0.05).

**Figure 3:**
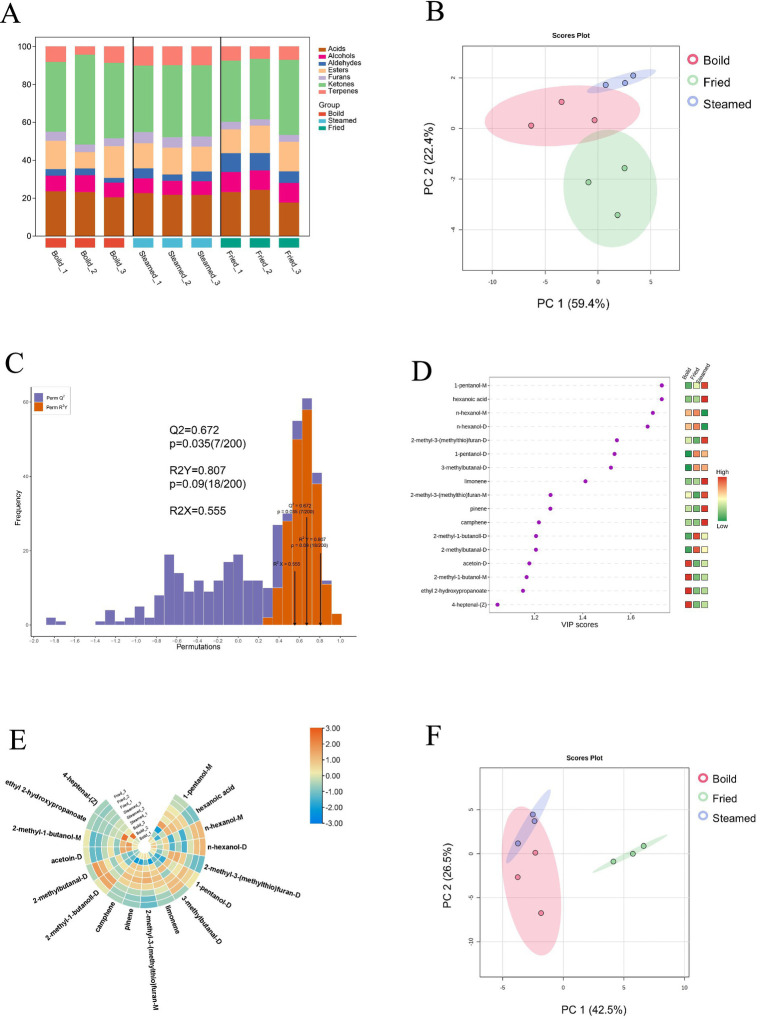
Chicken meatballs under different heating conditions: **(A)** proportion of volatile compounds in different groups; **(B)** PCA plot of all VOCs; **(C)** OPLS-DA model validation plot; **(D)** VIP values; **(E)** heatmap of differential compounds; **(F)** PCA plot of differential VOCs.

Figure 3A shows that ketones are the most concentrated compounds in each group, followed by terpenes and esters. These compounds generate a multitude of VOCs that contribute to the meaty aroma (44). The Maillard reaction varies with the type of meat (such as amino acid and glucose contents) and cooking conditions (including temperature and pH). It primarily produces volatile sulfur compounds, nitrogen-based heterocyclic compounds, and oxygen-containing heterocyclic compounds (45, 46).

Ketones contribute fruity aromas to food (44), and some volatile ketones are considered characteristic of fried food flavors (47). However, some studies suggest that volatile ketones may pose potential health risks (48). However, one is the most common substance across all the groups and belongs to the ketone class. It had the highest average content in the fried group and was markedly different (*p* < 0.05) (Table 2). This finding indicates that the frying method can increase the content of butanone. Moreover, acetoin is also present at high concentrations; it is a common food flavoring additive that is commonly present in nature. Microorganisms, plants, and animals can synthesize acetoin under certain conditions via various enzymes and pathways. It serves as a precursor to dozens of compounds and is characterized by its pleasant yogurt cream aroma and buttery fat flavor. Acetoin is a substance generally recognized as safe and is found in yogurt, fruits, vegetables, and flour (49). Terpenes are known for their fruity, floral, fresh, and spicy aromas (50), and their substantial presence in meat products often results from the use of spices, such as nutmeg, which contains various terpenes (51). Additionally, the production of terpenes in meat can occur in animals that consume green forage (52). Esters are known for their sweet taste and characteristic fruity aromas, which are formed by the esterification of acids and alcohols. In the process of heating meat products, fats are hydrolyzed into fatty acids, which then undergo oxidative degradation. This reaction produces short-chain fatty acids, alcohols, and esters (46). Ethyl acetate is also a high-content compound that we detected, being one of the most abundant esters in wine. It is a major factor in altering the typical sensory characteristics of wine and is also present in fruits and perfumes (53). On the basis of ANOVA (analysis of variance), we observed that the compound levels in the fried group were generally greater than those in the other cooking methods, reflecting the strong influence of cooking methods on the types and quantities of compounds in food (Table 2). These compounds are essential for the flavor characteristics of cooked meats because they add layers of complexity and richness to the aroma and taste.

#### 3.5.4 Multivariate statistical analysis of VOCs in Lueyang black-bone chicken meatballs

To determine the differences in the composition of VOCs in black-bone chicken meatballs prepared via different cooking methods, PCA was conducted (Figure 3B). PCA is an unsupervised technique for pattern recognition in multivariate data analysis (54). The respective percentages of each principal component represent how much of the total variance in the data it explains. Figure 3B indicates that PC1 and PC2 collectively explain 69% of the total variance. Additionally, OPLS-DA, a supervised statistical method for pattern recognition and data classification that is particularly effective with high-dimensional data, was employed. It is a variant of PLS-DA that incorporates orthogonal signal correction (55). *R_2_X* = 0.555 reflects the model’s explanatory power for the X matrix, *R_2_Y* = 0.807 represents the model’s explanatory power for the Y matrix, and *Q^2^* = 0.672 denotes its explanatory power for the Y matrix (Figure 3C). A *Q*^2^ > 0.5 is generally considered indicative of an effective model. In summary, these values suggest that the constructed model performs well in explaining variables and predicting outcomes (56).

Furthermore, to refine our understanding of which variables in the model have a decisive impact on the prediction outcomes, VIP values were used. VIP values measure the relative importance of each predictor variable within the model, helping us identify key variables that significantly contribute to the model’s response. A total of 17 compounds with VIP > 1 (*p* < 0.05) were selected (Figure 3D), including 1-pentanol-M, hexanoic acid, n-hexanol-M, n-hexanol-D, 2-methyl-3-(methylthio)furan-D, 1-pentanol-D, 3-methylbutanal-D, limonene, 2-methyl-3-(methylthio)furan-M, pinene, camphene, 2-methyl-1-butanoll-D, 2-methylbutanal-D, acetoin-D, 2-methyl-1-butanol-M, ethyl 2-hydroxypropanoate, and 4-heptenal-(Z). 1-Pentanol has the highest VIP value and is acknowledged as a strong candidate among renewable biofuels for diesel engine applications (57). It has been identified as a potential marker for distinguishing aromatic compounds between groups of water-boiled salted ducks (58) and braized chickens (59), significantly influencing the flavor characteristics of these foods. Visualizing the compounds with VIP > 1 via a heatmap allows for the observation of their relative abundance across different groups (Figure 3E). These compounds were subsequently subjected to two-dimensional PCA (Figure 3F), where the combined contribution of the first two principal components increased, resulting in an increase of 81.8%. This indicates that selecting compounds on the basis of the VIP eliminates variables that contribute less significantly or are irrelevant to the model, enabling the principal component analysis to more effectively capture and explain the variation among these key variables (60).

**Figure 4:**
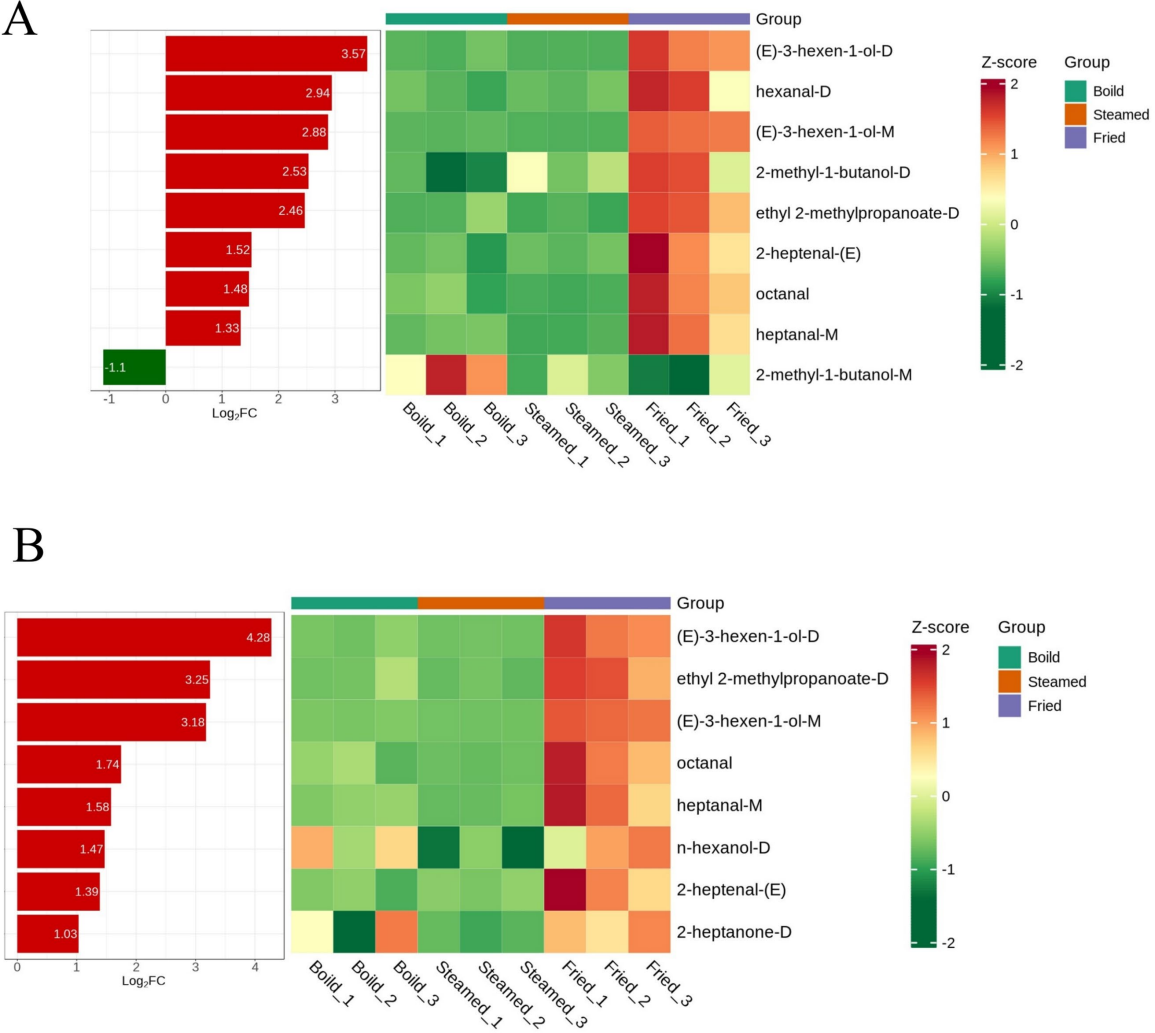
Identification of upregulated or downregulated substances with a threshold of log_2_FC > 1 or <−1 (*p* < 0.05) and VIP > 1: **(A)** boiled vs. fried and **(B)** steamed vs. fried.

Additionally, for these selected compounds with VIP > 1, pairwise comparisons between the three cooking methods were performed with a threshold of log_2_FC > 1 or < −1 (*p* < 0.05) to observe upregulated or downregulated substances, as shown in Figure 4A (boiled vs. fried) and Figure 4B (steamed vs. fried). Compared with those in the boiled group, octanal, 2-heptenal-(E), heptanal-M, (E)-3-hexen-1-ol-M, (E)-3-hexen-1-ol-D, hexanal-D, ethyl 2-methylpropanoate-D, and 2-methyl-1-butanol-M were upregulated in the fried group, whereas only 2-methyl-1-butanol-D was downregulated; compared with those in the steamed group, octanal, 2-heptenal-(E), heptanal-M, 2-heptanone-D, n-hexanol-D, (E)-3-hexen-1-ol-M, (E)-3-hexen-1-ol-D, and ethyl 2-methylpropanoate-D were upregulated. Among them, (E)-3-hexen-1-ol significantly changed. It belongs to the alcohol class and is a key VOC in plants. It is also an important active component contributing to the “green, grassy, and fresh” aroma of foods such as grapes and passion fruit. It is prevalent in fresh tea leaves and is the primary contributor to the green aroma of green tea (61, 62). Interestingly, when the boiled and steamed groups were compared, only one compound, n-hexanol-D (log_2_FC < −1), was downregulated. This may be due to frying promoting the formation of more flavor compounds, as high temperatures accelerate chemical reactions, especially the Maillard reaction (45). Frying provides a dry (or nearly dry) cooking environment that helps quickly dehydrate the food surface and create a crust, thereby locking in the internal flavor compounds and reducing compound loss. In contrast, boiling and steaming methods are more similar; both are conducted in water or steam, which can dissolve and carry away some water-soluble compounds. Moreover, these two methods involve cooking at a lower temperature, typically approximately 100°C, which is limited by the boiling temperature of the water, resulting in the formation of fewer VOCs. Although the VOCs of Lueyang black-bone chicken meatballs prepared via different cooking methods were analyzed, comprehensive aroma profiling requires further investigation via GC-O and complementary techniques.

## 4 Conclusion

This study conducted a comparative assessment of Lueyang black-bone chicken meatballs prepared via three different cooking methods. The findings indicated that air-fried meatballs received the highest sensory ratings. Air-frying notably increased color parameters (a, b) as well as textural attributes, including hardness, cohesiveness, and chewiness (*p* < 0.05). A total of 42 VOCs, including aldehydes, alcohols, esters, ketones, terpenes, furans, and acids, were identified. The fried samples presented a distinct VOC profile, with significant enrichment of key aroma compounds such as (E)-3-hexen-1-ol (fruity, green), ethyl 2-methylpropanoate (sweet, alcoholic, and ethereal), octanal (fatty), heptanal-M (oil, green), and 2-heptenal-(E) (fatty, fruity) (VIP > 1, *p* < 0.05, Log2FC > 1). These findings suggest that air-fried chicken meatballs possess superior quality attributes, offering valuable insights for the development and commercialization of ready-to-eat Lueyang black-bone chicken meatball products. Further investigations into advanced glycation end products, heterocyclic amines, and detailed aroma profiles during air-frying will be presented in future studies.

## Data Availability

The original contributions presented in the study are included in the article/Supplementary material, further inquiries can be directed to the corresponding authors.

## Footnotes

^1^ https://www.biodeep.cn/home/tool
