# Supplementary material for: Comparative analysis of the quality characteristics and flavor volatiles of Lueyang black-bone chicken meatballs cooked via different methods
Source: Front Nutr. 2025 Jul 23;12:1629738. doi: 10.3389/fnut.2025.1629738 (PMC12325199; doi:10.3389/fnut.2025.1629738)
Supplement: Supplementary file 1 [file Table_1.DOCX]

Table S1 Sensory evaluation criteria

| Category | Scoring Criteria | Score Range |
| --- | --- | --- |
| Color (25 points) | Uniform appearance with gloss | 19-25 |
|  | Slightly uneven appearance with gloss | 12-18 |
|  | Uneven appearance without gloss | 5-11 |
| Taste (25 points) | Has inherent chicken aroma, juicy and refreshing, full aftertaste, lingering fragrance | 19-25 |
|  | Meat taste, average fragrance, no off-flavor | 12-18 |
|  | Lacks chicken aroma, has off-flavor | 5-11 |
| Texture (30 points) | Refreshing, tender, crispy, moderate hardness | 21-30 |
|  | Somewhat crispy, fairly fine, soft | 11-20 |
|  | Texture too hard or too soft, lacks resilience, sticky | 1-10 |
| Juiciness (20 points) | Fresh and juicy product | 15-20 |
|  | Product is somewhat dry, less juicy | 10-14 |
|  | Product too dry, no juice | 5-9 |

Table S2 Sensory evaluation results of Lueyang black-bone chicken

| Sample | Color and Appearance | Texture | Score |
| --- | --- | --- | --- |
| Boiled | Uneven color and poor gloss; meat taste and fragrance are average with a slight off-flavor. | Moderate texture, resilient; refreshing and moderately juicy. | 69 |
| Steamed | Slightly uneven color, glossy; meat taste and fragrance are average, no off-flavor. | Moderate and fine texture, resilient; refreshing and moderately juicy. | 77 |
| Fried | Uniform color, glossy; has inherent chicken aroma, full aftertaste, lingering fragrance. | Refreshing, tender, crispy, moderate hardness; product is fresh and juicy. | 89 |
